# Nosocomial Achromobacter xylosoxidans Infection Presenting as a Cavitary Lung Lesion in a Lung Cancer Patient

**DOI:** 10.7759/cureus.9818

**Published:** 2020-08-17

**Authors:** Vinoja Sebanayagam, Paul Nguyen, Mo'ath Nassar, Ayman Soubani

**Affiliations:** 1 Pulmonary, Critical Care and Sleep Medicine, Wayne State University School of Medicine, Detroit, USA; 2 Internal Medicine, Wayne State University School of Medicine, Detroit, USA

**Keywords:** achromobacter xylosoxidans, lung cavity, pneumonia

## Abstract

*Achromobacter xylosoxidans* is a Gram-negative bacillus that is known to cause nosocomial infections, primarily in patients with hematological malignancies. The most common primary manifestation is bacteremia. We report a novel case of primary *A. xylosoxidans* infection presenting as a cavitary lung lesion with associated pneumonia in a lung cancer patient who showed no evidence of malignant disease progression after radiation therapy. Our patient was initially admitted for acute hypoxic respiratory failure requiring mechanical ventilation. Initial computed tomography (CT) revealed a cavitary lesion in the right upper lobe of the lung. Diagnostic bronchoscopy with bronchoalveolar lavage (BAL) was performed and was negative for infectious etiologies including tuberculosis (TB) and fungal infections. Cytology was also negative for malignancy. However, the bacterial culture grew *A. xylosoxidans*. Antimicrobial therapy was initiated based on culture susceptibilities and the patient showed significant improvement in oxygen requirements. Due to poor functional status, the palliative care route was pursued and mechanical ventilation weaning was not performed. Cavitary pulmonary infections secondary to *A. xylosoxidans* are rarely reported in the medical literature. After conducting a thorough PubMed database search of the medical literature, we believe this is the first case of *A. xylosoxidans* infection manifesting as a cavitary lung lesion with associated pneumonia in a lung cancer patient.

## Introduction

*Achromobacter xylosoxidans* is an aerobic, non-fermenting Gram-negative bacillus that is capable of oxidizing xylose [1,2]. It is known to cause healthcare-associated infections, predominantly in immunocompromised individuals [1]. Within this group of individuals, it has been more commonly seen in patients with hematological malignancies [3-6]. The most common clinical presentation appears to be primary bacteremia, followed by pneumonia, and catheter-associated bacteremia [7]. Other reported presentations include meningitis, cellulitis, pyelonephritis, and endocarditis [1,7,8].

Cavitary lung lesions are typically observed in a variety of diseases, including infections, malignancies, and autoimmune diseases [9]. The most commonly reported infectious etiologies include pulmonary tuberculosis (TB), common bacteria such as *Staphylococcus Aureus,* and fungi such as aspergillosis [9,10]. Cavitary lung lesions caused by *A. xylosoxidans* are extremely rare in the medical literature [11]. The case report we found in the literature was a middle-aged immunocompetent man who had a community-acquired *A. xylosoxidans *induced cavitary lung lesion that responded to antibiotics [11]. However, to the best of our knowledge, there are no reports in the literature of *A. xylosoxidans *associated cavitary lung lesions in lung cancer patients. Therefore, we report a novel case of *A. xylosoxidans* manifesting as a cavitary lung lesion in a lung cancer patient who did not show signs of cancer progression after radiation therapy.

## Case presentation

**Figure 1:**
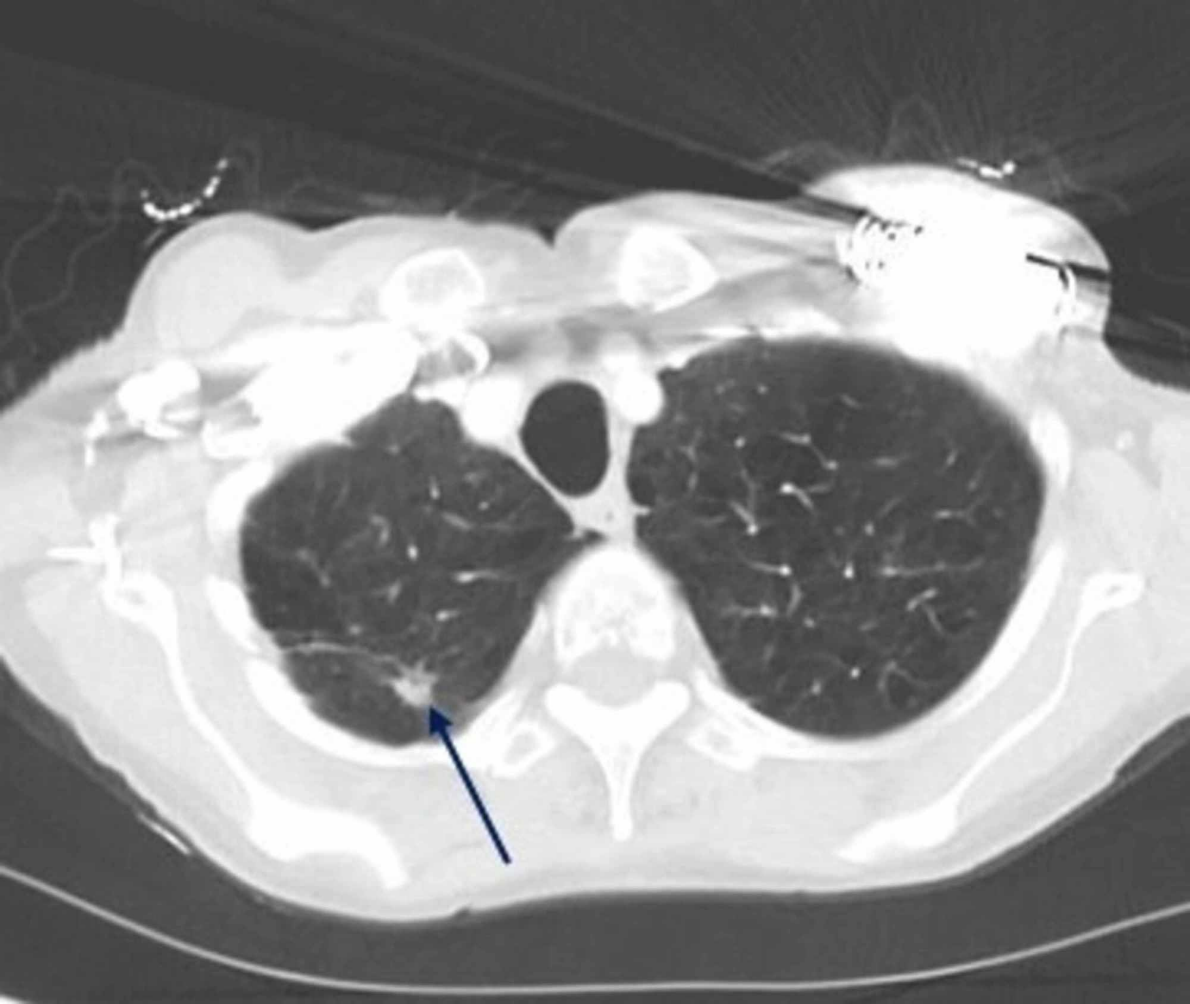
CT-thorax showing a right upper lobe malignant lung nodule (blue arrow), after radiation therapy

A 69-year-old woman with a past medical history of chronic obstructive pulmonary disease and stage IA2 squamous cell carcinoma of the right upper lobe of the lung was diagnosed with the malignancy one year prior to the current hospital admission. The patient was treated with stereotactic body radiation therapy (SBRT) to the right upper lobe, due to poor performance status and advanced chronic obstructive pulmonary disease. Computed tomography of the chest (CT-thorax) performed three months prior to the current admission showed significant improvement in the size of the malignant lesion (Figure 1). In the current admission, the patient presented to the hospital with shortness of breath and was admitted to the intensive care unit (ICU) for the management of acute hypoxic respiratory failure requiring invasive mechanical ventilation. Initial diagnostic workup including complete blood count, basic metabolic panel, and microbiological studies was unremarkable.

**Figure 2:**
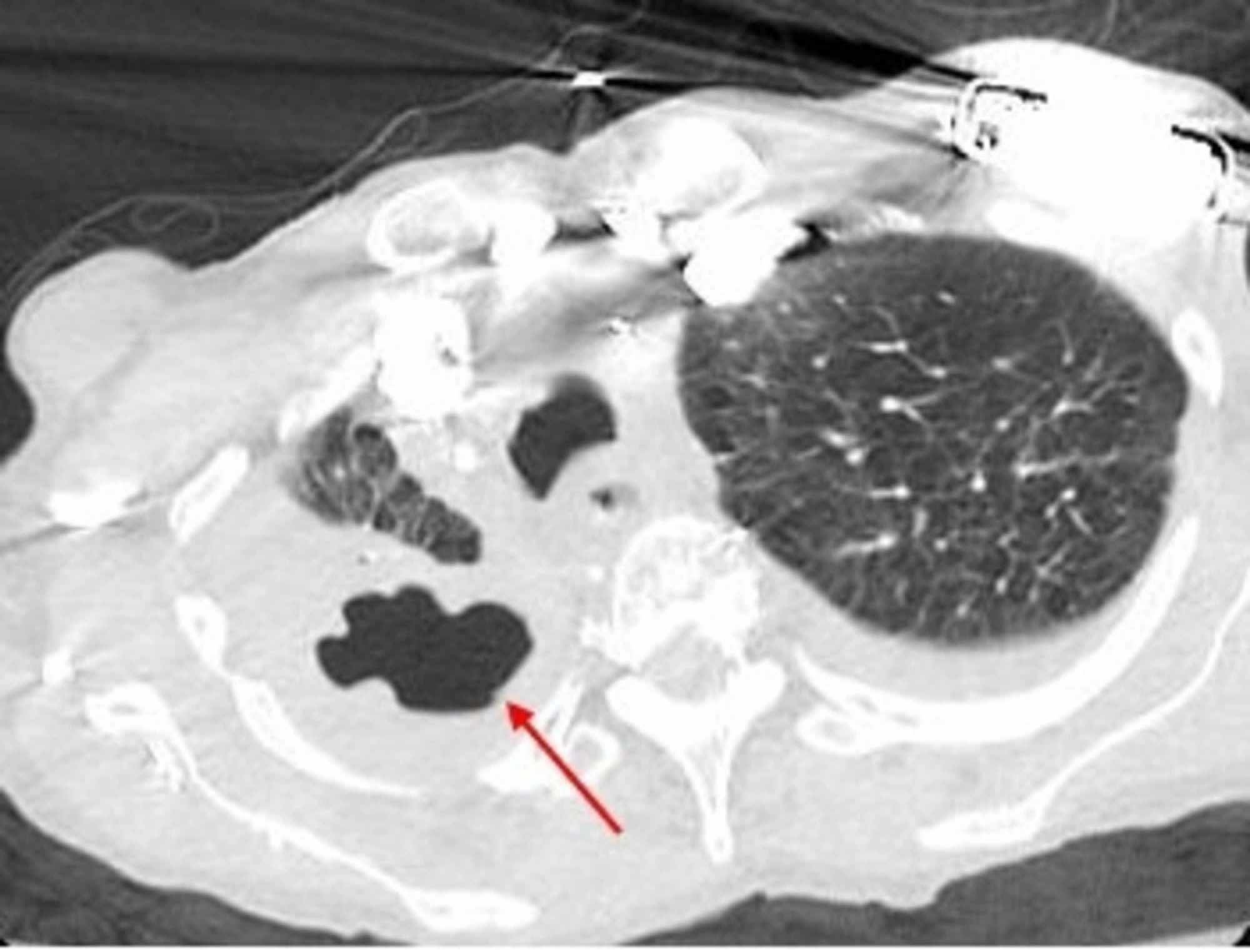
CT-thorax obtained at admission showing a gas-filled cavitary lesion (red arrow) surrounded by consolidation, in the upper lobe of the right lung

A CT-thorax revealed extensive airspace consolidation in the right upper lobe of the lung with a 3.8 cm gas-filled irregular cavity surrounded by consolidation (Figure 2). Broad spectrum antimicrobial therapy was initiated and included imipenem. 

Diagnostic bronchoscopy with bronchoalveolar lavage (BAL) was performed and resulted in the growth of *A. xylosoxidans* on microbiological culture (Table 1 shows antibiotic sensitivity testing). The BAL was negative for TB or fungal infections. The cytological analysis revealed no evidence of malignant cells. 

**Table 1:** Antibiotic sensitivity results BAL: bronchoalveolar lavage, MIC: minimum inhibitory concentration; S: sensitive; R: resistant; MS: moderately sensitive.

Microorganism: A. xylosoxidans in the BAL		
Antibiotic	MIC (µg/mL)	Interpretation
Amikacin	>32	R
Aztreonam	>16	R
Cefepime	16	MS
Ceftazidime	8	S
Ceftriaxone	>32	R
Ciprofloxacin	>2	R
Gentamicin	>8	R
Imipenem	2	S
Piperacillin/Tazobactam	≤2/4	S
Tobramycin	>8	R
Trimethoprim/Sulfamethoxazole	≤0.5/9.5	S

**Figure 3:**
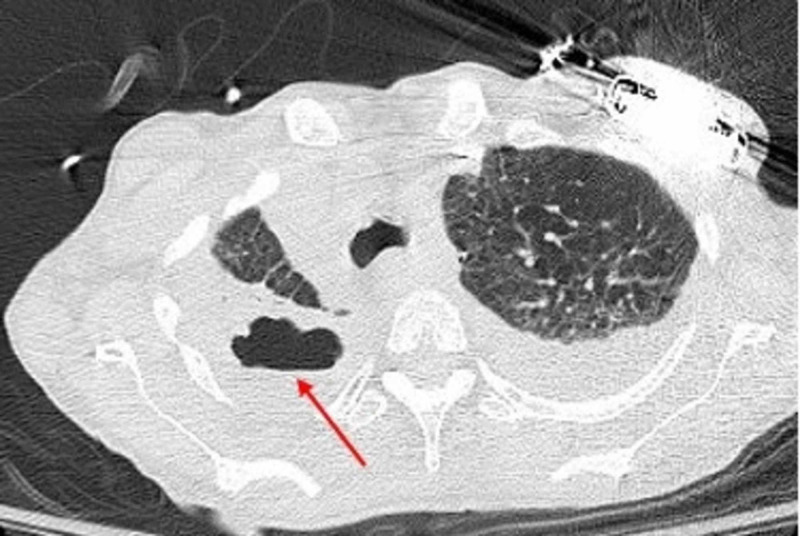
CT-thorax obtained one week after treatment initiation showing stable right upper lobe cavitary lesion (red arrow) with surrounding consolidation

CT-thorax was repeated one week after initiation of treatment and showed stable cavitary lesion (Figure 3). Oxygen requirement improved significantly and weaning of mechanical ventilation was achieved. However, re-intubation was performed due to excessive secretions and inability to protect airways. After goals of care discussion, the patient and family decided to pursue palliative care.

**Figure 4:**
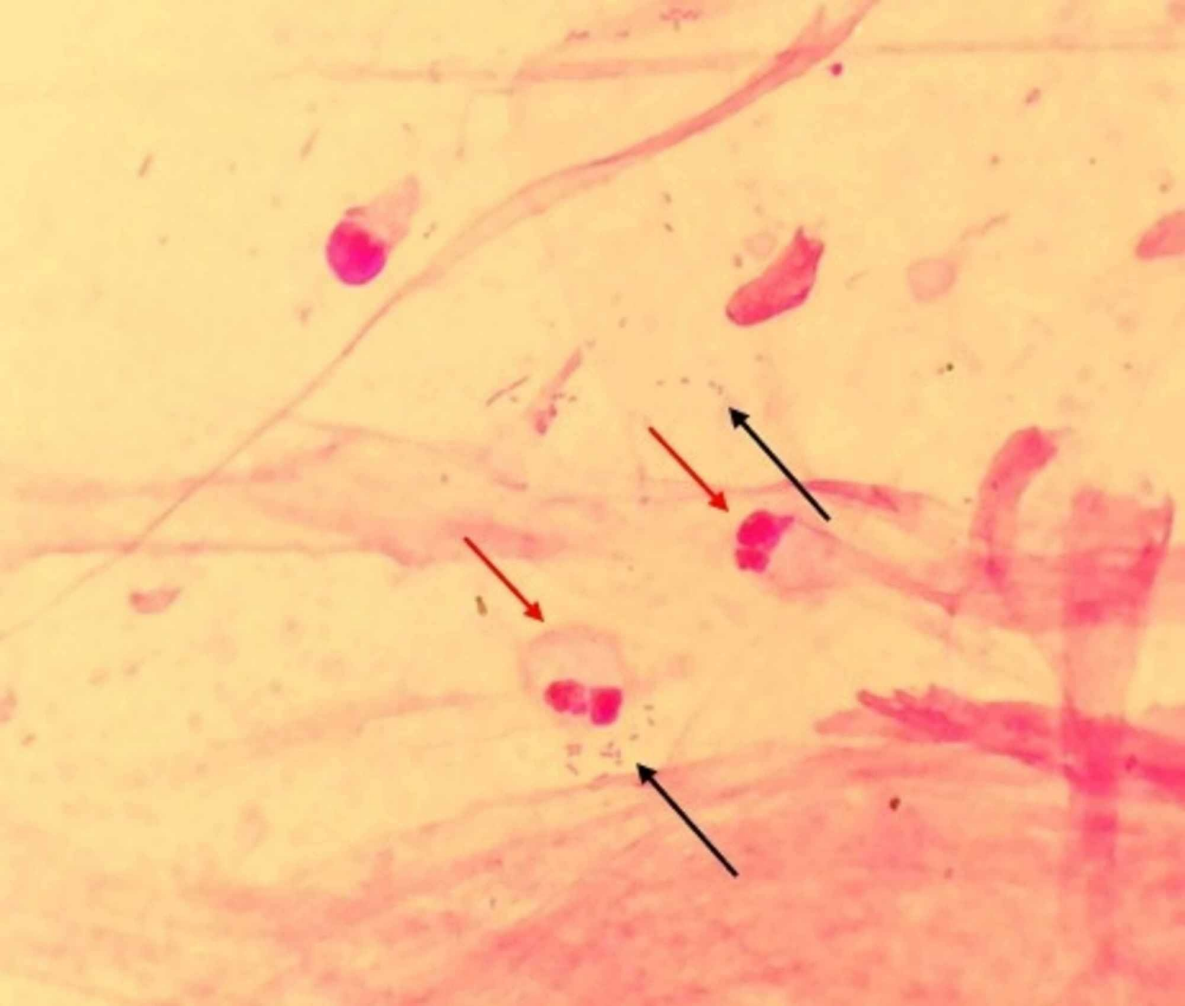
Gram stain image showing Gram-negative A. xylosoxidans (black arrows) with numerous neutrophils (red arrows)

A repeat sputum culture obtained 2.5 weeks after the bronchoscopy, continued to be positive for *A. xylosoxidans* susceptible to imipenem (Figure 4 is an image of the Gram stain from our patient). 

## Discussion

*A. xylosoxidans* was first described by Yabuchi et al. after it was isolated from the ear discharge of patients with chronic otitis media [2]. It is widely present in aqueous environments including well water and swimming pools [12]. However, it is also known to be a nosocomial colonizer that has been isolated from solutions found in the healthcare setting, including dialysis solutions, and chlorhexidine gluconate solutions, as well as fomites such as mechanical ventilators, intravenous catheters, and urinary catheters [12]. *A. xylosoxidans* is an opportunistic pathogen that primarily affects patients with underlying medical issues such as malignancy (30%), cardiac disease (21%), and immunosuppression (27%) [12]. It primarily presents as uncomplicated bacteremia, but has also caused pneumonia, soft tissue infections, and urinary tract infections among others, and has been isolated from multiple sources including blood, cerebrospinal fluid, sputum, skin, etc. [12]. We were able to find only one case during our literature search, in which this bacteria presented as a cavitary lung lesion [11]. However, this presentation is different from our case in that it stemmed from a community-acquired infection in an otherwise healthy immunocompetent man who had no significant past medical history or recent hospitalizations [11].

The two main differential diagnoses for cavitary lung lesions in adults are malignant tumors and benign infections [9]. Our patient has a history of squamous cell cancer, presenting as a right upper lobe nodule which subsequently decreased in size after radiation therapy. Figure 1 shows the CT-thorax performed three months prior to the current hospital admission, demonstrating the right upper lobe malignant nodule. Bronchial wash cytologic analysis from the BAL was negative for malignant cells. While it has low sensitivity for cancer diagnosis [13], it is unlikely that the malignancy had progressed significantly over the course of three months. This makes infectious etiology more likely especially in the light of positive BAL culture. Just three days prior to the current admission, our patient had been discharged after being admitted and treated for acute encephalopathy secondary to a urinary tract infection. Given that *A.xylosoxidans* is a well-established nosocomial colonizer, it is very likely that the bacterial inoculation occurred during her recent hospital stay. Furthermore, significant improvement in oxygen requirement was noted upon treatment with appropriate antimicrobial therapy during the current admission.

From a treatment standpoint, consistent with previous antibiotic susceptibility profiles reported in the literature [1], *A. xylosoxidans* isolated from our BAL sample was susceptible to imipenem. Based on in vitro susceptibility tests reported in the literature, empirical antibiotic coverage that includes a carbapenem agent is usually adequate to treat *A. xylosoxidans* infections [1]. Imipenem is known to have adequate lung penetration [14]. Overall, our patient received empiric imipenem coverage for a total of eight days with an improvement in oxygen requirements. However, no significant improvement in the size of the cavitary lesion was noted on the imaging done a week after initiation of treatment. Optimally, significant changes in chest imaging are noted six to eight weeks after completion of therapy; however, this was not appropriate for our patient as she pursued palliative care. Of note, the repeat sputum culture obtained 2.5 weeks after the bronchoscopy was positive for *A. xylosoxidans*, reinforcing the notion that the bacterium served as a primary causative agent of infection rather than just a contaminant. The persistence of the bacterium in the respiratory culture could be attributed to the inadequate duration of antibiotic coverage, as the bacterium isolated from the sputum culture was still susceptible to imipenem.

## Conclusions

In this case report, we present a case of *A. xylosoxidans* infection that manifested as a single cavitary lung lesion in the setting of pneumonia, in a lung cancer patient who showed no radiographical signs of cancer progression after radiation therapy. The novelty of this case lies in the way this bacterium presented in our patient. We present the first case of a nosocomial *A. xylosoxidans* infection manifesting as a single cavitary pulmonary lesion in a lung cancer patient.
